# Reflections on beam configuration optimization for intensity-modulated proton therapy

**DOI:** 10.1088/1361-6560/ac6fac

**Published:** 2022-06-27

**Authors:** Wenhua Cao, Humberto Rocha, Radhe Mohan, Gino Lim, Hadis M Goudarzi, Brígida C Ferreira, Joana M Dias

**Affiliations:** 1 Department of Radiation Physics, The University of Texas MD Anderson Cancer Center, Houston, United States of America; 2 University of Coimbra, CeBER, Faculty of Economics, Coimbra, Portugal; 3 University of Coimbra, INESC Coimbra, Coimbra, Portugal; 4 Department of Industrial Engineering, University of Houston, Houston, United States of America; 5 Institute of Biophysics and Biomedical Engineering, Faculty of Sciences, University of Lisbon, Lisbon, Portugal

**Keywords:** beam angle optimization (BAO), proton therapy, IMPT, RBE, robustness

## Abstract

Presumably, intensity-modulated proton radiotherapy (IMPT) is the most powerful form of proton radiotherapy. In the current state of the art, IMPT beam configurations (i.e. the number of beams and their directions) are, in general, chosen subjectively based on prior experience and practicality. Beam configuration optimization (BCO) for IMPT could, in theory, significantly enhance IMPT’s therapeutic potential. However, BCO is complex and highly computer resource-intensive. Some algorithms for BCO have been developed for intensity-modulated photon therapy (IMRT). They are rarely used clinically mainly because the large number of beams typically employed in IMRT renders BCO essentially unnecessary. Moreover, in the newer form of IMRT, volumetric modulated arc therapy, there are no individual static beams. BCO is of greater importance for IMPT because it typically employs a very small number of beams (2–4) and, when the number of beams is small, BCO is critical for improving plan quality. However, the unique properties and requirements of protons, particularly in IMPT, make BCO challenging. Protons are more sensitive than photons to anatomic changes, exhibit variable relative biological effectiveness along their paths, and, as recently discovered, may spare the immune system. Such factors must be considered in IMPT BCO, though doing so would make BCO more resource intensive and make it more challenging to extend BCO algorithms developed for IMRT to IMPT. A limited amount of research in IMPT BCO has been conducted; however, considerable additional work is needed for its further development to make it truly effective and computationally practical. This article aims to provide a review of existing BCO algorithms, most of which were developed for IMRT, and addresses important requirements specific to BCO for IMPT optimization that necessitate the modification of existing approaches or the development of new effective and efficient ones.

## 1. Introduction

Intensity-modulated proton therapy (IMPT), ostensibly the most powerful form of proton radiotherapy, was first introduced in the late 1990s by Lomax (1999). It has become widely available and the dominant mode of proton therapy over the last decade (Kooy and Grassberger 2015, Mohan and Grosshans 2017). IMPT employs multiple proton beams incident from judiciously selected directions. Each beam is composed of a large number of narrow pencil beams (i.e. ‘beamlets’) having a sequence of appropriate energies. These beamlets are delivered using magnetic scanning technology. The intensities of the beamlets of all beams are optimized simultaneously using sophisticated mathematical algorithms to produce dose distributions that optimally balance target coverage and the sparing of organs at risk (OARs). In contrast with photon intensity-modulated radiation therapy (IMRT), IMPT has an extra degree of freedom, that of energy, which allows it to achieve significantly superior therapeutically effective dose distributions.

There are multiple forms of IMPT (Lomax 1999). In its two-dimensional form, single-field uniform dose IMPT, also called single-field optimized IMPT, or SFO (Zhu et al 2010), the intensities of the beamlets of each beam are individually optimized to deliver a uniform dose to the target and minimize dose outside the target; however, the role of other beams is ignored in the process. This technique is the simplest but cannot achieve the optimality of other approaches. In the distal edge tracking form of IMPT, spots (the terminal ends of beamlets) are placed at the distal edge of the target volume. This technique has the drawback of producing dose distributions that are highly sensitive to sources of uncertainties such as interfractional and intrafractional changes in anatomy. In the three-dimensional mode of IMPT, spots of beams incident from various directions are typically arranged uniformly in the target volume, and their intensities are optimized simultaneously. 3D-IMPT has been shown to have several advantages over the other techniques mentioned and is currently the most prevalent approach. 3D-IMPT is the focus of this article, and, for brevity, we will simply call it IMPT hereafter.

In the current practice of IMPT planning, the number of beams and their angles (i.e. beam configurations) are chosen subjectively based on prior trial-and-error experience and practical considerations (e.g. efficiency of quality assurance and treatment delivery). The number of IMPT beams is usually 2 or 3 and rarely more than 4 or 5. Occasionally, for some disease sites, non-coplanar beams are also used. The optimal choice of beam configuration can have profound impact on the quality of dose distributions and on patients’ outcomes.

Primarily because of the high dose gradients at the end of the range of protons, proton therapy is more sensitive than photon therapy to variations in anatomy, which can introduce significant uncertainty in the dose delivered compared to what is seen on the treatment plan (Lomax 2008a, 2008b, Mohan and Sahoo 2015, Mohan and Grosshans 2017, Mohan *et al* 2017). IMPT is particularly sensitive to uncertainties, since the highly heterogeneous dose distributions of each constituent beam, when combined, fit like a 3D jigsaw puzzle to produce the desired uniform dose distribution in the target volume while maximally sparing normal tissues. In the face of uncertainties, this fit may be lost, meaning that the dose distributions delivered to both the target and normal tissues may be quite different from what was planned. To address this problem, in the current practice of proton therapy, the directions most affected by anatomic changes are avoided; however, this may lead to directions that may be suboptimal clinically. For prostate cancer, for instance, 2 lateral parallel opposed proton beams are most commonly used. They are not necessarily optimal from a dosimetric or clinical perspective, but they avoid paths that may be affected by stomach and bladder filling or weight gain or loss over the course of radiotherapy. To render IMPT resilient in the face of uncertainties, techniques have been developed for robust optimization (RO) of IMPT plans and for the robustness evaluation of proton dose distributions (Lomax 2008a, 2008b, Albertini et al 2010, 2011, Liu et al 2012a, 2012b, Casiraghi et al 2013, Liu et al 2013, 2016, Placidi et al 2017, Zaghian et al 2017, Ge et al 2019). The choice of beam configuration can affect the robustness of dose distributions, and beam configuration optimization (BCO) criteria should consider robustness.

In the current practice of proton therapy, the relative biological effectiveness (RBE) of protons relative to photons is generally assumed to be a constant of 1.1. In reality, the RBE is a complex function of linear energy transfer (LET), dose, tissue and/or cell type, and endpoint (Mohan *et al* 2017, Mohan and Grosshans 2017). Unforeseen toxicities and suboptimal response to proton therapy, compared to expectations, have been observed (Peeler et al 2016, Mohan *et al* 2017, 2018, Haas-Kogan et al 2018), which may in part be attributable to the assumption of a constant value for proton RBE. Such outcomes are prompting the development of new variable RBE models and an increasing acceptance of their use in IMPT optimization. Rørvik et al (2018) reviewed eleven published phenomenological RBE models that include multiple physical and biological factors influencing RBE, concluding that different models lead to considerably different results for the RBE estimations and RBE-weighted doses. Some in the field have preferred to use LET as a surrogate for RBE in optimization criteria (Deng et al 2021, Unkelbach et al 2016, Cao et al 2018). In either case, the RBE at the distal edges of proton beams can be significantly higher than 1.1. Without special measures, high-RBE regions, which are often in normal tissues distal to the target to ensure adequacy of target coverage, can contribute to normal tissue injury. The choice of beam configuration is often influenced by the need to minimize biologically effective dose (or LET in high-dose regions) in normal tissues. Alternatively, IMPT optimization and BCO criteria may incorporate LET or variable RBE to mitigate toxicity in regions distal to the target.

Recently, another potential benefit of proton therapy has emerged, that of sparing the immune system. Such sparing is presumably attributable to the compactness of proton therapy dose distributions, i.e. the significantly smaller low and intermediate ‘dose bath’. Conventional radiotherapy, for instance with IMRT (or its newer cousin, volumetric modulated arc therapy), produces a large dose bath and can greatly deplete circulating lymphocytes, immune cells that are highly sensitive to radiation. This can lead to severe lymphopenia, which has been shown to be strongly associated with poor outcomes (Yovino and Grossman 2012, Tang et al 2014, Grossman et al 2015, Wild et al 2015). The smaller dose bath of protons spares lymphocytes, which can mitigate immune suppression and lead to superior outcomes compared to IMRT (Shiraishi et al 2018, Ebrahimi et al 2019, van Rossum et al 2020, Mohan et al 2021). IMPT may be able to spare lymphocytes to a greater degree compared to passively scattered proton therapy (Routman et al 2019, Kim et al 2021). Moreover, it has been hypothesized that IMPT, when optimized based on criteria that include sparing of circulating lymphocytes and immune OARs, can further spare lymphocytes without compromising target coverage or exceeding conventional normal tissue dose constraints. Beam configurations can play an important role in such optimization because, for instance, using shorter paths to the target may reduce the dose bath, and the choice of angles may affect sparing of immune OARs (e.g. bone marrow, thymus, lymph nodes, spleen, heart).

Thus, BCO, which is critical for achieving the most clinically effective dose distributions, must take into consideration LET or variable RBE, the sensitivity of dose distributions to uncertainties, and the sparing of the immune system. In each of these aspects, IMPT significantly differs from IMRT. In addition, typical IMRT plans may have a much larger number of beams—anywhere from 5 to 11 beams, often spaced equiangularly. This may mean that the optimality of IMRT dose distributions is less dependent on beam configurations. On the other hand, the number of adjustable variables available in IMPT is at least an order of magnitude greater because of the additional dimension of energy. Therefore, in principle, the number of beams required for IMPT can be much smaller. Another consideration specific to IMPT, which does not apply to IMRT, is the limit on the minimum monitor units (MUs) per spot owing to the inability of the beam monitoring system to detect extremely low values.

In short, BCO techniques developed for IMRT may not be directly extensible to IMPT. However, considering the special requirements of proton therapy listed in the previous paragraphs and detailed throughout this article, it makes sense here to briefly revisit previously developed beam angle optimization techniques, even though the overwhelming majority have been applied to IMRT only. In fact, searching MEDLINE/PubMed for the terms ‘BCO’ or ‘Beam angle optimization’ in conjunction with ‘IMRT’ yielded 130 publications in the past 10 years (2011–2021), while the corresponding search with ‘IMPT’ yielded only 22 even without any time limit. Scrutinizing each of these 22 articles closely, the list of IMPT articles that actually focus on beam angle optimization reduces to just 5 articles (Cao et al 2012, 2015, Gu et al 2018, 2019, 2021). Note that beam angles strategically selected as done by Kirk et al (2017) or comparison of different beam angle configurations as done by Feng et al (2020) or by van de Schoot et al (2016) cannot be considered beam angle optimization. The goal of our report is not to carry out a systematic review of either BCO or IMPT, but rather present authors’ experience and reflections shedding light on the challenges, opportunities and potential research directions on BCO for IMPT.

The paper is organized as follow. In sections 2 and 3 below, currently available intensity distribution (i.e. fluence map) optimization and BCO approaches are reviewed, and their limitations at meeting the unique requirements of IMPT are discussed. Section 4 summarizes the current state of the art and defines a research roadmap for the future. (Note that we use the terms ‘fluence map optimization’ [FMO] and intensity distribution optimization interchangeably in this review).

## 2. BCO algorithms

BCO, which involves beam angle optimization as well as the determination of the optimal number of beams, is a highly nonconvex mathematical problem. The space of solutions can be extremely large and full of ‘local minima traps’, which can make it very difficult to find the globally optimal solution. This section discusses commonly used criteria and algorithmic approaches for FMO and BCO.

### 2.1. Optimization criteria

The mathematical optimization is based on specified criteria to guide the search for the optimal solution. These criteria are specified by an objective function, a mathematical formulation of the treatment planning directives. The optimization algorithm repeatedly computes the value of the objective function, i.e. the score, during the iterative search process. The goal is to minimize the score, which, effectively, represents how far the solution is from the directives.

A simple example of an objective function is the sum of variances of dose in each of the structures of interest (tumor or a critical normal tissue). Each variance term is assigned an appropriate weight depending on the relative clinical importance of the structure. If the requirement is to constrain only a subvolume of a structure to within a specified dose limit, i.e. to impose a dose-volume constraint, only a subset of points in the structure volume violating the constraint are considered in computing the score. The goal is to bring the required number of such points into compliance. A given structure may have multiple dose-volume constraints. Examples of additional objective functions, including their IMPT-specific extensions (e.g. for biological and RO) are described in appendix A.

**Figure 1.**
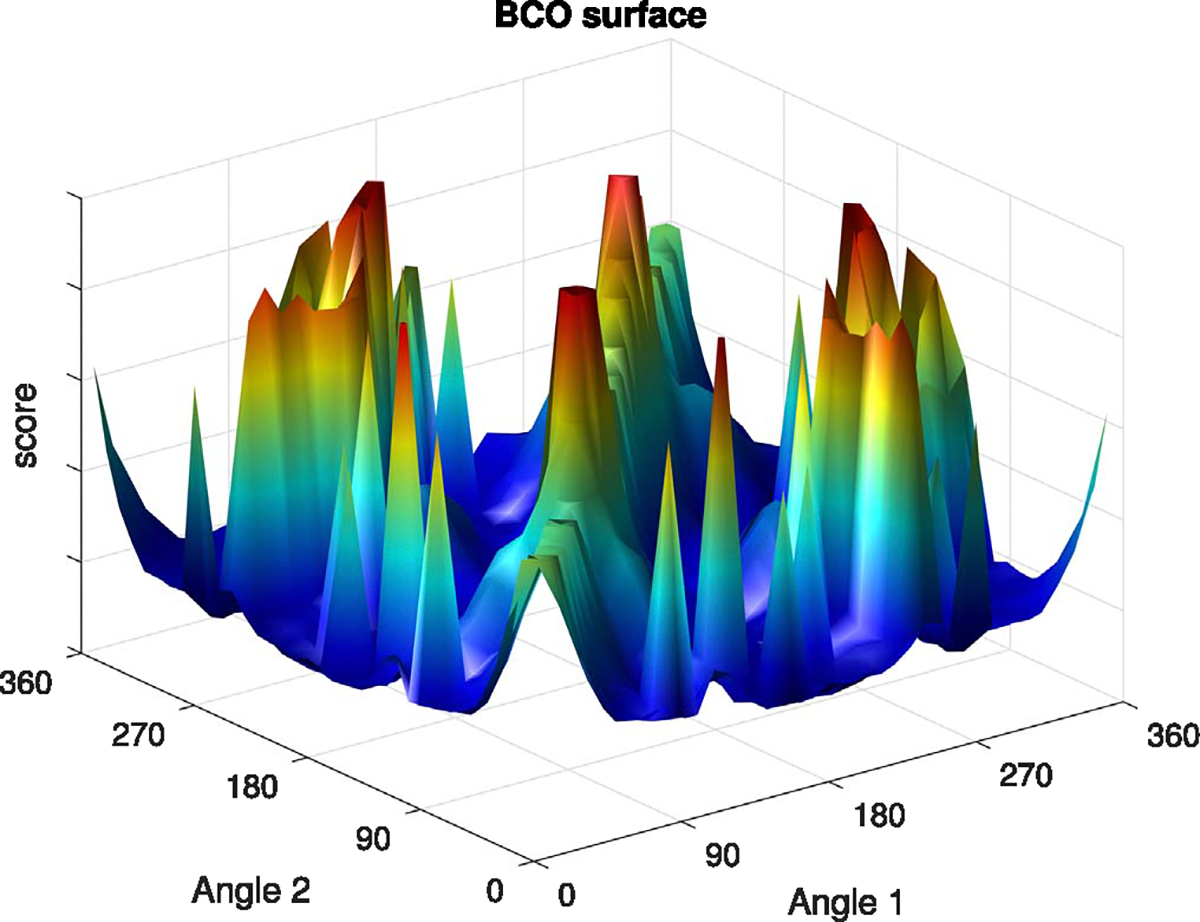
BCO search surface illustrating many local minima traps for a prostate cancer case using a coplanar 2-beam configuration. Finding the global minimum is a difficult task because each of these valleys corresponds to a local minimum where a BCO algorithm may get trapped.

Owing to the so-called ‘curse of dimensionality’ (Chen et al 2015), the complexity of the search space in terms of its size and the number of local minima increases exponentially with increasing number of degrees of freedom. In general, for BCO, this complexity depends partly on the optimization criteria and partly on the number of beams as well as the extent of the search domain. The optimization process may inadvertently get trapped in one of the local minima that may be significantly inferior to the globally optimal solution. In theory, the best optimization algorithm would be expected to converge to a high-quality solution, avoiding the local minima traps. The challenges of exploring the BCO search space are illustrated in figure 1 for a simple prostate cancer case with a 2-beam coplanar configuration. The figure depicts the score value for all possible pairs of beams comprising the searchable surface. Even for only 2 beams, there is a large number of valleys, i.e. local minima traps.

### 2.2. Optimization algorithms

#### 2.2.1. Optimization of the number of beams

Most of BCO approaches assume that the number of beams is decided *a priori*, considering mainly the disease location and existing OARs. More beams can improve OAR sparing whilst guaranteeing a proper tumor coverage, at the cost of increasing total treatment delivery time and even increased leakage radiation due to the MUs increase. This means that a larger number of beams should only be considered if it leads to a better dose distribution. The optimization of the number of beams can be done in a variety of ways. One is brute-force: simply find the best beam configuration for a different number of beams, and then compare these optimal treatment plans and choose the one that is considered as the best. One example of such strategy can be found in Liu et al (2006). Another possibility is to integrate the optimization of the beam directions with the decision on the optimal number of beams by considering a greedy and sequential choice of beams. One beam is chosen at a time, until a newly added beam does not improve the plan quality. This strategy has been adopted for IMRT BCO (Breedveld et al 2012, Bangert and Unkelbach 2016). This approach significantly reduces the number of beam combinations to be explored (Bangert et al 2013). However, being a ‘greedy’ approach by adding the beam that, at that point, contributes most to score minimization, a near-optimal beam configuration may be missed since the search space is truncated whenever the direction of the newly added beam is fixed. Metaheuristics are a possible alternative for the simultaneous optimization of the directions and the number of beams (Schreibmann et al 2004). It is possible to represent a treatment plan by two different vectors: one vector that represents beam intensities and another binary vector, with one element for each possible beam, that is equal to one if that beam is used and equal to zero otherwise (Lee et al 2006). Machine learning based approaches can also be used to determine an optimal number of beams, assuming an informative dataset is available, since this problem can be interpreted as a classification problem (Dias et al 2013).

Proton arc therapy (PAT) has already been proposed as the next step in proton therapy, similar to the evolution of IMRT to VMAT. If this is the case, it could be thought that BCO would not be important for proton therapy anymore. Actually, as happens with VMAT, BCO can still be used for defining VMAT control points and trajectories in order to achieve efficient delivery while maintaining dosimetric benefits (Carrasqueira et al 2021), so it is expected that a similar thing will happen with PAT. The benefits of PAT over IMPT are still to be determined. It is possible that the radiobiological aspects of PAT could lead to an improved therapeutic index (Carabe-Fernandez et al 2020, Li et al 2021).

#### 2.2.2. Optimization of beam directions

Most published BCO algorithms were developed for IMRT. The fact that IMPT BCO needs fewer beams than does IMRT is an advantage from the computational point of view. This means that some algorithmic approaches that were found to lead to unreasonably high computational times for IMRT may be acceptable for IMPT. However, simple exhaustive searches of all possible beam angle combinations (Wang et al 2004) are still expected to lead to unacceptably large computation times. In any case, despite the differences in requirements for photons and protons, the BCO algorithms used for photons can serve as an important starting point for protons.

At the simplest level of BCO methods is the seminal work of Goitein et al (1983) on beam’s eye view (BEV), which is used to select the best beam directions considering the anatomical relationships between the target volume and critical normal structures. BEV has been a popular approach to address BCO considering geometric criteria only (Lu et al 1997, Pugachev and Xing 2001); however, it ignores dosimetric requirements. On the other hand, optimal beam directions based on dosimetric criteria are generally counter-intuitive (Stein et al 1997). Nevertheless, an automated BEV approach can complement advanced dosimetric-based BCO approaches by limiting the search space to regions that minimize the overlap of sensitive structures with the target, reduce the effects of anatomy changes, and shorten the beam paths.

It is important to note that different beam configurations can lead to very different optimum beamlet intensities and dose distributions. The best results are expected when both FMO and BCO are performed together. Typically, BCO approaches require reoptimization of fluence maps and recomputation of the dose distributions for each beam configuration tested, which adds immensely to the computational burden, especially when attempting to achieve a global optimum in the presence of many local minima traps. Global optimization algorithms, well established in other scientific areas, have been proposed for BCO. Examples include simulated annealing (Djajaputra et al 2003, Dias et al 2015), neural networks (Rowbottom et al 1999), genetic algorithms (Li et al 2004, Dias et al 2014), particle swarm optimization (Li et al 2005), integer programming algorithms (Lee et al 2003), and response surface algorithms (Aleman et al 2009). Although such global strategies can reduce the probability of being trapped in local minima, the globally optimal solution cannot usually be guaranteed in a clinically acceptable computational time (Lim et al 2014b).

Local optimization algorithms have also been proposed for BCO, including neighborhood search, i.e. searching around the best solution found so far (Aleman et al 2008, Mišić et al 2010, Lim and Cao 2012); cluster analysis, i.e. grouping of beam ensembles (Bangert and Oelfke 2010); and gradient search, i.e. using objective function derivatives to guide the search (Craft 2007). These algorithms need less computational time, but the quality of their solutions is very sensitive to the initial beam configuration (Lim and Cao 2012). Hybrid approaches combining global and local methods have also been proposed in which a global strategy is used to identify one or a small number of initial configurations for further optimization with a local strategy, thereby achieving a high-quality, though not necessarily globally optimal, solution in acceptable computational times. Lim et al (2014b), for instance, proposed a 2-phase hybrid approach in which a good feasible solution is quickly found in the first phase by using a global strategy, which then serves as the starting point for a neighborhood search to find a locally optimal solution. Pattern search methods also combine global and local features (Rocha et al 2013a, 2016, 2019). Pattern search methods are organized around 2 steps. In the first search step, any global strategy may be used to explore the entire search space, providing a global perspective. In the second step, an improvement of the solution found so far is assured by following specific defined and promising search directions (Alberto et al 2004). Different global search strategies have been applied as components of pattern search methods (Rocha et al 2013a, 2013b).

BCO search space can be explored in a continuous or discretized manner. In the latter, only discretized beam directions are considered. Even for a small space of such beams (e.g. 36), the BCO combinatorial formulation would lead to a large, computationally demanding optimization problem (Bangert et al 2012).

The time for computation of dose distributions plays an important role in choosing the optimization approach. Unfortunately, computational times are not always reported in the published literature, or the time for pre-calculation of dose distributions is not included in the total computational time. This makes comparisons of the reported computational performances of the different algorithms difficult or biased (Haas et al 1998, Rowbottom et al 1999, Li et al 2004, Rocha et al 2013a, Dias et al 2015). Computational times can also be influenced by the criteria used. For instance, optimization based on anatomical criteria to choose the initial configuration would lead to shorter computation times. On the other hand, multicriteria optimization methods (Breedveld et al 2012) (see next section) can be extremely demanding. Even the choice of the radiotherapy treatment planning system (TPS) or the resolution of the planning CT scans influences computational performance. Table B1 in appendix B shows different BCO approaches mentioned, including a qualitative comparison of their computational performances, acknowledging the aforementioned limitations.

Recently, machine learning approaches have been proposed for BCO. One approach considers the use of neural networks to rapidly calculate good approximations of dose distributions (Dias et al 2014, Sadeghnejad Barkousaraie et al 2020). The main advantage of this approach is the attractive computation time. However, the validity of such an approach would need to be tested against methods that employ accurate dose computation techniques.

As mentioned above, although theoretically BCO algorithms previously developed for IMRT can be adapted for IMPT, doing so is not straightforward. For instance, the robustness of IMPT dose distributions, which plays a crucial role in the choice of beam configurations, must be considered. In some of the proposed algorithms, FMO, BCO, and RO are integrated (Cao et al 2012, Gu et al 2019). Robustness can also be handled within the FMO portion of the BCO loop (Taasti et al 2020). This means that any mainstream global optimization algorithm, e.g. genetic algorithms, may continue to be used in FMO and BCO (Seo et al 2020). Another challenge is that LET or variable RBE also need to be explicitly incorporated in the BCO. This has recently been addressed in a publication by Gu et al (2021). One topic that has not been adequately addressed is the simultaneous optimization of beam orientations and spot patterns, instead of in two levels. This is due to the computational complexity of the problem despite recent efforts to develop computationally efficient algorithms (Gu et al 2018, Sadeghnejad Barkousaraie et al 2020).

We should note that some current commercial TPSs offer rudimentary BCO tools. An example is the Plan Geometry Optimizer, also called Beam Angle Optimizer, of Varian’s Eclipse TPS, which iteratively optimizes gantry angles, with presumed benefits over the manual selection of beams (Srivastava et al 2011). However, the scarcity of published results on BCO performance from solutions provided by commercial TPSs is an indication that they are not widely used in clinical practice, either because they are still time consuming or because they have not yet convinced practitioners of their benefits. To the best of our knowledge, commercial TPSs offer no effective BCO solution for IMPT.

## 3. IMPT-Specific considerations of relevance to BCO

Both FMO and BCO for IMPT have some special considerations attributable to the unique characteristics of protons. These are discussed in the subsections below.

### 3.1. Robust optimization

There are a set of treatment-related uncertainties that should be explicitly considered when planning a treatment. In conventional radiotherapy, important uncertainties come from interfractional and intrafractional anatomy changes, or setup variations. In proton therapy, proton range uncertainties are also a reality. These different sources of uncertainty can be responsible for severe heterogeneity, degradation, and misalignment of dose contributions from often highly modulated beams. In conventional radiotherapy, these treatment-related uncertainties are usually incorporated into margins assigned to clinical target volumes to define the planning target volumes. However, the planning target volume concept has several limitations for proton therapy, especially for IMPT. For instance, proton range uncertainties cannot be adequately addressed by margins alone. It is possible to generate treatment plans that are resilient in the face of such factors by using RO techniques (Unkelbach et al 2018). Implicitly, RO reduces gradients in dose distributions, rendering them less sensitive to sources of uncertainties.

Several different approaches for RO have been proposed. Most of them have focused on minimizing the impact of systematic or interfractional range uncertainties and setup errors on IMPT dose distributions, i.e. 3D RO. More recently, methods incorporating temporal effects (e.g. intrafractional respiratory motion) have also been studied with 4D RO. Regardless of the RO approach, the key point is that the beam configuration can significantly affect the robustness of dose distributions. For instance, beams that pass through anatomies that are likely to change will lead to less robust dose distributions. Directions of such beams will be avoided by BCO that incorporates robustness in its optimization criteria. We note that an alternative to the use of BCO to avoid passage through structures more likely to change is the use of adaptive strategies such that treatment plans could be modified on a nearly daily basis to accommodate interfractional changes. However, such strategies may be highly resource intensive and cannot account for the impact of intrafractional uncertainties. Many efforts have already been done in this direction (Albertini et al 2020, Paganetti et al 2021).

RO, especially 4D RO, may increase the computational burden significantly. Robustness criteria may be integrated into BCO, or, alternatively, BCO may be performed first and RO then performed for the resulting optimized beam configuration. The latter is likely to be computationally more efficient. However, whether such a sequential optimization approach is dosimetrically equivalent to simultaneous optimization is unknown and needs to be investigated. Another question that needs to be answered is about the interaction between BCO and RO. If RO reduces dose gradients, does it render local neighborhood search unnecessary due to the fact that dose distributions will become insensitive to small changes in beam angles?

The following paragraphs briefly summarize some of the common RO approaches. *A priori*, it would seem that the relation between BCO and any of the approaches would be the same; however, this needs to be confirmed with future research.

#### 3.1.1. Deterministic RO

Treatment uncertainties can be formulated in a deterministic fashion for RO. One way is to minimize the score for the worst-case scenario among a set of uncertainty scenarios. An example is a set of 9 independent scenarios obtained by 6 shifts along +/− orthogonal axes, maximum and minimum of proton range, and 1 nominal scenario. In this approach, FMO and BCO would aim to minimize the score of the worst among the 9 scenarios in each iteration (Liu et al 2012b). Several variations of the worst-case approach have been evaluated. Fredriksson and Bokrantz (2014) concluded that no method outperformed the others under all conditions, whereas Casiraghi et al (2013) found that, in general, the worst-case scenario approach is likely to underestimate the maximum dose error in voxels near density heterogeneities.

#### 3.1.2. Probabilistic RO

In probabilistic RO, each uncertainty component, e.g. proton range or patient position, is considered as a random variable and assigned a probability distribution. The robustness is incorporated into the objective function using the expected value of the voxel dose. An example of probabilistic approach is a study by Unkelbach et al (2009), which used a stochastic gradient descent algorithm to solve probabilistic RO. Voxel sampling and scenario sampling techniques were used to estimate the gradient of the objective function during optimization iterations.

#### 3.1.3. Minimization of maximum (or worst-case) regret

Another possibility, already successfully tested in other fields, is the use of relative robustness. Instead of focusing on the worst-case only, the solution is obtained by minimizing the ‘maximum regret’, considering the entire set of scenarios. Regret is defined, for each scenario, as the difference between the score of the solution in the current search iteration and the score of the optimal solution based only on that scenario. In a first stage, the optimal solution for each scenario s, i.e. the minimum score $\left(S_{s}^{*}\right)$, is calculated independently, i.e. ignoring other scenarios. Here the asterisk denotes the optimum. As each scenario has different characteristics, the optimal solution, in general, will be scenario dependent. In other words, for n scenarios, there will be n different optimal solutions with different optimal score values $S_{s}^{*}$. The objective is to calculate a single solution that minimizes the worst regret. Regret, as defined above, for scenario s would then be the difference between $\left(S_{s}\right)$, the score during the iterative optimization, and its optimal solution score $\left(S_{s}^{*}\right)$ for scenario s. The process of minimization of regret iteratively computes $\left(S_{s}-S_{s}^{*}\right)$ differences to find the solution that reduces the maximum regret to a minimum value. Implicitly, this means that the final solution will not necessarily be optimal for any one of the scenarios but is expected to be a well-behaved solution that is balanced among all scenarios. An example of minimizing the ‘maximum regret’ is provided in appendix C.

#### 3.1.4. Multicriteria optimization

It is particularly useful to use RO as a part of multicriteria optimization methods. Multicriteria optimization, which is normally used to interactively trade off dosimetric requirements among volumes of interest (tumor and normal tissues), may also be used to trade off robustness versus dosimetric requirements. Chen et al (2012), for instance, incorporated robustness into a multicriteria optimization framework and developed an efficient approach to generate Pareto surface plans for treatment planners to choose the most desired one. Kamal Sayed et al (2020) take advantage of the fact that, for the same beam configuration, different scalarization parameters for the fluence optimization will lead to different Pareto points and present a bilevel optimization model that builds a Pareto front with contributions from different beam configurations. However, it is not *a priori* obvious how multicriteria optimization and RO could jointly be integrated with BCO, a topic that needs further research.

#### 3.1.5. 4D robust optimization

Respiratory motion in thoracic or liver cancer patients can lead to a range mismatch. In addition, interplay between dynamic beamlet delivery and intrafractional respiratory motion may seriously affect the quality of dose distributions requiring 4D RO methods (Liu et al 2016). Approaches to minimize the impact of intrafractional motion on dose distributions and robustness include definition of a range-adapted internal target volume, breath-hold, gating, tumor tracking, and 4D treatment planning. Engwall et al (2018) describe a 4D RO approach that includes the time structures of the delivery and organ motion to generate plans that are robust against interplay effects. The authors assume that the delivery is synchronized with the patients breathing. The assumption of perfect synchronization can be, however, difficult to achieve clinically. Liu et al (2016) combined 4D treatment planning with RO to create a robust 4D optimization method for 9 uncertainty scenarios for each of the 10 respiratory phases without the requirement of perfect synchronization. Another study using a similar approach also reported promising advantages of 4D RO compared to conventional optimization and 3D RO (Ge et al 2019). On the other hand, Knopf et al (2022) present a review of RO approaches that consider breathing motion represented by a set of scenarios via multiple image sets, and conclude that the clinical necessity of 4D RO has not yet been demonstrated. While 4D RO reduces the sensitivity of dose distributions to respiratory motion, the relationship between respiratory motion and beam configurations is not yet totally clear, other than that BCO may avoid directions that are most affected by motion. However, Liu et al (2016) concluded that the explicit modeling of respiratory motion resulted in a treatment plan where the beams that played the major role where the ones parallel to the tumor motion direction. Similarly, Feng et al (2020) conclude that the SI oblique posterior beams are better than the RL oblique posterior beams for the distal esophageal carcinoma treatments. In any case, developing 4D RO BCO methods should lead to an automatic choice of the best directions whenever respiratory motion amplitudes are significant.

### 3.2. LET and RBE-weighted dose optimization

Currently, IMPT optimization (i.e. FMO) is based on biologically effective dose distributions assuming a fixed RBE of 1.1 for protons. However, as mentioned above, the RBE is a complex nonlinear function of dose, LET, tissue, cell type, and other factors, and it is increasingly acknowledged that the traditional assumption of fixed proton RBE needs to be abandoned. IMPT optimization techniques based on criteria that employ variable RBE-weighted dose (*D*_*v*RBE_), computed with one of the many available models (Wilkens and Oelfke 2004, McNamara et al 2015, Stewart et al 2018) have been reported. However, there are concerns about uncertainties in the predictions of these models. Therefore, an alternative has been proposed in which terms based on LET, assuming it to be a surrogate for biological effect, are incorporated in the optimization criteria. LET, being a physical quantity, can be calculated accurately (Wilkens and Oelfke 2003, Grassberger and Paganetti 2011). However, there is an ongoing debate about the use of current RBE models versus LET in optimizing and evaluating proton therapy plans.

During the iterative FMO process, beamlet intensities change with each iteration, resulting in corresponding changes in dose and LET values. In *D*_*v*RBE_-based optimization, biologically effective dose in each iteration is calculated using one of the models. In LET-based optimization, the LET is used directly or the RBE-weighted dose is assumed to be a simple function of dose and LET, for instance *D*_*v*RBE_ = *D*_physical_(1 + λ × LET), where λ is an empirically determined parameter. An example of a LET-based approach is a 2-step method in which an initial IMPT plan based on physical (or assuming RBE = 1.1) dose is optimized (Unkelbach et al 2016). Then, a prioritized optimization scheme, in which physical dose objectives are constrained to values close to those of the initial plan, is used to optimize LET. Liu et al (2020) describe a somewhat different LET-guided RO approach, where LET and physical dose distributions are simultaneously taken into consideration in the objective function value. Their approach was tested for head-and-neck cancer. Other approaches incorporating LET into IMPT optimization criteria have also shown promising results (Wan Chan Tseung et al 2016, An et al 2017, Cao et al 2018, Traneus and Oden 2019).

An important goal of LET or RBE-weighted dose distribution optimization is to divert high-LET protons away from high-dose normal tissue regions into the tumor target. The relevance of these techniques to BCO is that certain beam configurations may make achievement of this goal more likely because RBE increases as a function of depth and is highest near the end of the proton range. Therefore, beam directions sought by the BCO would be those that minimize the exposure of critical normal tissues with distal edges of the beamlets.

**Figure 2.**
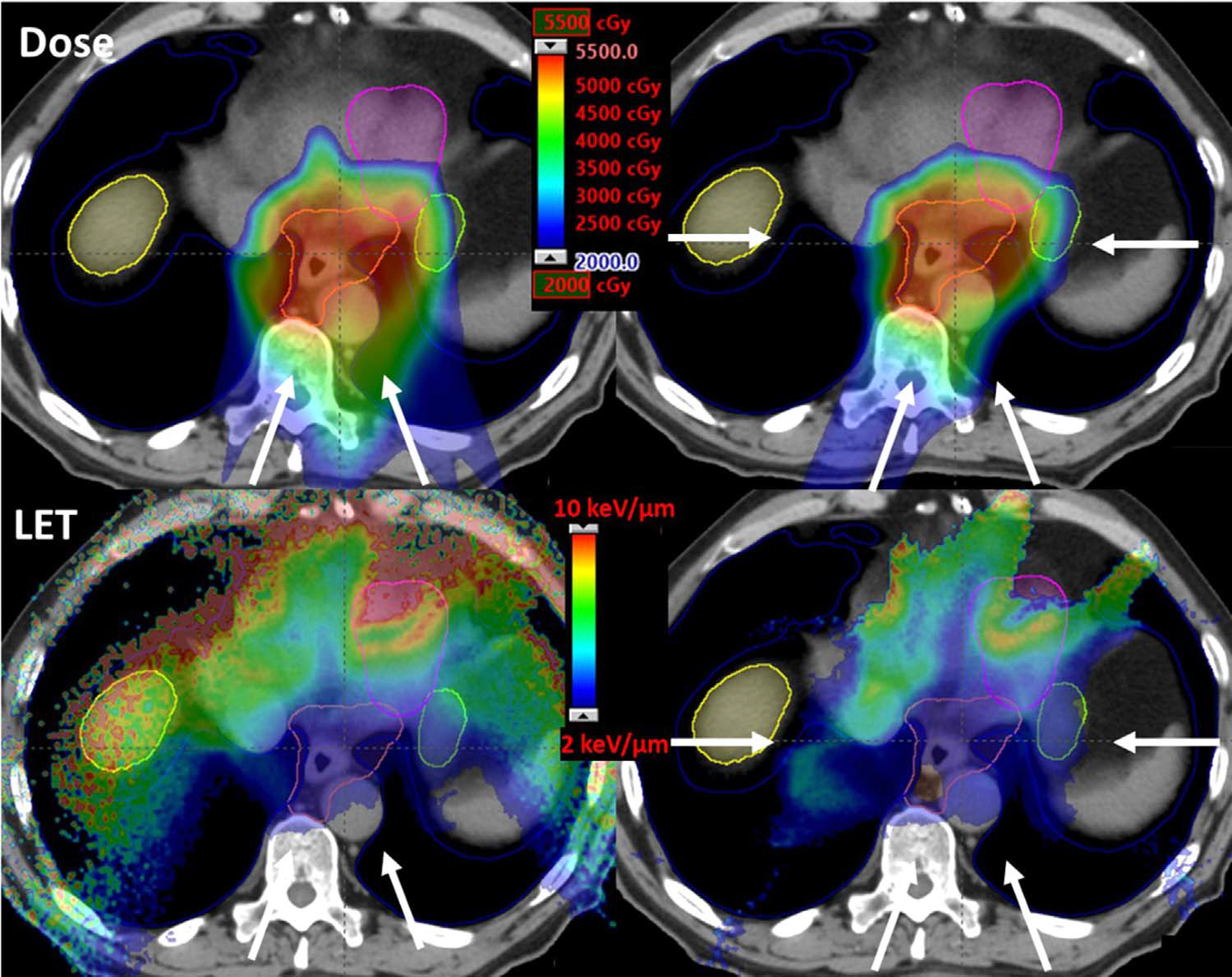
Comparison of dose and LET (calculated using dose-averaged LET) distributions between 2-beam (left column) and 4-beam (right column)IMPT plans for an esophageal cancer patient. Both plans were optimized using conventional IMPT optimizer to achieve similar physical dose distributions(first row). However, the 4-beam plans reduced the LET hotspots in normal tissues including heart, liver, and stomach (second row). Dose and LET were calculated by an in-house Monte Carlo simulation system. White arrows indicate beam directions.

Figure 2 is an example comparing 2 different beam configurations for an esophageal cancer case. The IMPT plan with a 2-beam configuration, both posterior obliques, would be appropriate to achieve sufficient target coverage with good sparing of normal tissues if RBE = 1.1 is assumed. However, this beam configuration would result in high LET, and therefore high variable RBE-weighted dose, in regions located beyond the distal edge of the beams. By adding 2 lateral beams, the 4-beam configuration plan shows reduced dose-averaged LET. In this example, beam configurations were not optimized. It simply emphasizes the importance of BCO in that different beam configurations can lead to significantly different LET distribution in normal tissues.

### 3.3. Maximizing the sparing of the immune system

The large, low-radiation ‘dose bath’ in traditional photon-based therapy can deplete highly radiosensitive circulating lymphocytes, resulting in lymphopenia, a common side effect of conventional radiotherapy (Yovino and Grossman 2012, Tang et al 2014, Grossman et al 2015, Wild et al 2015). Severe lymphopenia (grade 3 or higher) has been associated with adverse treatment-related outcomes. Recently, it has been demonstrated that proton therapy, owing to its compact dose distributions (smaller dose bath), has the potential to significantly mitigate lymphopenia and improve survival. Research is ongoing to determine predictors of severe lymphopenia, to develop predictive models, and to use these models to define dosimetric constraints on immune OARs and the dose bath. Such constraints, along with standard constraints on normal tissues, may be incorporated into FMO and BCO for potential improvement in outcomes.

The relevance of BCO to sparing of the immune system is that, in general, specific beam configurations may be preferable for sparing the immune system, specifically for reducing the dose bath. For instance, beams with shorter distances to the target and plans employing a smaller number of beams would be more effective in reducing the dose bath; however, this must be balanced against sparing of critical normal tissues and ensuring target coverage.

### 3.4. Treatment deliverability constraints

Typically, there is a threshold of the minimum number of MUs per beamlet for a scanning proton beam system, which ensures detectability of beamlets by the monitoring system. Postprocessing procedures are usually performed on optimized plans to enforce deliverable MU constraints. If small spot spacings are required to increase target dose homogeneity or to lower the OAR dose, such a procedure may cause a significant dose distribution deviation between the optimized and postprocessed deliverable plans (Zhu et al 2010). The feasibility of enforcing MU constraints within optimization has been studied using constrained linear programming (Cao et al 2013) and quadratic programming approaches (Shan et al 2018). However, it is still unclear how these approaches affect plan quality if different planning criteria (e.g. robustness and LET/RBE) are used or different tumor sites are tested. Similarly, it is not clear what role deliverability constraints would play in BCO. In general, a smaller number of beams would minimize the impact of constraints on minimum MUs per spot. An alternative would be to minimize the number of spots as a part of FMO and BCO. Another approach, that should be investigated in the future, may be to first proceed with FMO and BCO optimization, ignoring deliverability constraints, and then perform FMO again, this time taking deliverability constraints into account.

## 4. Summary and future directions for BCO

Over the last few decades, radiation oncology has seen considerable advances that offer an increased range of treatment options to patients with cancer. Motivated by the advantageous dosimetric characteristics of proton beams, the clinical use of proton therapy, especially IMPT, has been increasing rapidly. IMPT has specific features that make approaches developed for IMRT not directly transferable to IMPT. In the context of the present report, BCO approaches for IMPT will require considerable further research and development to address the numerous issues and unanswered questions raised above.

One such issue is the higher sensitivity of IMPT dose distributions to unavoidable uncertainties. As discussed in section 3.1, several RO approaches have been developed for IMPT. However, their integration into BCO is challenging, in part due to their very high computational burden. Multiple strategies to achieve clinically practical methods are possible but need to be investigated and compared. For instance, it is not clear whether FMO and BCO, when integrated with RO, should be performed simultaneously, or whether it would be sufficient to first perform FMO and BCO followed by RO based on the optimal beam configuration. The latter approach may be computationally much more practical. The extremely high computational resource requirement of comprehensive FMO and BCO could also be addressed by the use of graphics processing units and high-performance clusters.

Similarly, challenges related to integration of LET or RBE-weighted dose, immune sparing with IMPT, and incorporation of treatment delivery constraints in BCO remain to be addressed. Moreover, an important question that needs to be answered is whether BCO can lead to significantly superior biologically effective and robust dose distributions. In other words, can we make BCO and FMO, integrated with all the factors considered in section 3—namely robustness, LET (or *D*_*v*RBE_), sparing of the immune system, and adhering to delivery constraints—produce treatment plans that are clinically more effective than those achieved without BCO?

There is now a growing interest in applying machine learning and artificial intelligence techniques to radiation treatment planning (Jarrett et al 2019, Vandewinckele et al 2020). It is quite conceivable that machine learning will be highly useful in defining optimal treatment objectives and expected outcomes for each patient. However, it is not so clear what machine learning can achieve for treatment plan optimization, or specifically for BCO. Machine learning techniques can certainly be used to accelerate proton dose computations for FMO and BCO. In principle, machine learning techniques could also be used to identify optimal beam configurations, at least for an initial guess or the definition of a smaller search space, to be fine-tuned by BCO. However, a large training dataset of optimal beam configurations would be required, which is not currently available. One can foresee that, over time, the use of IMPT incorporating BCO would lead to the collection of sufficiently large datasets of high-quality plans appropriate for machine learning and artificial intelligence applications.

## 5. Conclusions

In conclusion, BCO for IMPT is more critical, but at the same time more complex, than for IMRT. RO as a part of BCO is essential to ensure not only that beam directions are chosen such that regions of significant anatomic change are avoided, but also that all other IMPT-related uncertainties are considered in FMO. LET and variable RBE must also be considered in BCO so that beam directions that lead to high biologically effective dose in critical organs are avoided. BCO should also lead to beams that minimize irradiation of circulating lymphocytes and immune OARs while adhering to standard-of-practice tumor dose and normal critical-structure constraints. Moreover, BCO must ensure adherence to deliverability constraints.

Finally, the state of the art of IMPT is continually evolving. For instance, there is ongoing research to develop personalized, as opposed to population-averaged, models that can more accurately predict tumor response and normal tissue toxicities personalized to a given patient’s baseline characteristics and dose distribution patterns. FMO and BCO plans optimized based on such models should be able to deliver high-quality treatments tailored to each specific patient’s characteristics and produce predictable outcomes. Another evolving area is that of improving the efficiency of IMPT planning. A totally automated IMPT procedure should be capable of calculating robust plans in short computation times. However, the high computational burden will very likely necessitate the use of graphics processing units or high-performance computer clusters for routine clinical implementation.

## Appendix A. Example of objective functions

For dose distribution optimization, the objective function usually consists of a weighted sum of individual objectives, at least one for each anatomic structure of interest (tumor or normal tissue). The weights (or penalties) are used to specify each individual objective’s relative importance. In addition, ‘hard’ or ‘soft’ constraints may be imposed, guaranteeing that the calculated solutions are within certain boundaries. Certain beam directions may be forbidden, while others may be preferred. The BCO problem may, for example, be formulated as

$$\underset{x}{min}\;f(D)=\sum_{m}\;\left(\alpha_{T_{m}}\sum_{i\in T_{m}}\;\frac{{\left(D_{i}-{\overset{‾}{D}}_{l}\right)}^{2}}{\left|T_{m}\right|}\right)+\sum_{n}\;\left(\alpha_{S_{n}}\sum_{i\in S_{n}}\;\frac{{\left(D_{i}-{\overset{‾}{D}}_{l}\right)}^{2}}{\left|S_{n}\right|}\right)+\sum_{a}\;\alpha_{a}g\left(x_{a}\right)\;\;\text{subject}\;\text{to}\;D=Ix\;\;x_{j,a}⩾0,j\in J,a\in A;.$$

The variable $x_{j,a}$ is the intensity of beamlet j from beam a, and the matrix I is the dose influence matrix containing dose values in all voxels from all beamlets per unit intensity, which are used to calculate dose distribution D. The function $g\left(x_{a}\right)$ is a function of the beamlet intensities of beam a and used to control the number of beams, e.g. an $L_{2}$ norm of $x_{a}$. The parameter α indicates the priority of each objective, while $T_{m}$ and $S_{n}$ contain voxels of target m and normal structure n, respectively.

The objective function should drive the iterative process towards solutions that comply with the planning directives defined by clinicians, but it does not have to incorporate those exact values. For instance, if achieving $D_{95}$ equal to 66 Gy proves to be hard, then the parameters associated with this individual objective can be different from 66 Gy ($\overset{-}{D_{l}}$ can take higher values, implicitly increasing the importance of this structure in the optimization). Such adjustments may also be automated, by dynamically adjusting penalties or dosimetric objectives so that the clinical directives are fulfilled. Multi-criteria optimization is an alternative to the use of a single weighted objective function. Different plans are calculated by changing the importance given to each of the individual objectives, and the principle of ‘Pareto optimality’ is then used to graphically select the preferred dose distribution.

## Appendix B. Supplementary table

**Table B1.** Summary of different BCO algorithms.

	Approach	Algorithm	Criteria	Computation time	Proton suitability	Reference (s)

**Optimization**	Global	Exhaustive search	Anatomical	++++	+++	Goitein et al (1983), Lu et al (1997)
			Dosimetric	+	+	Wang et al (2004)
		Simulated annealing	Dosimetric	+	++	Dias et al (2015), Djajaputra et al (2003)
		Genetic algorithms	Dosimetric	+	+	Dias et al (2014), Li et al (2004)
		Particle swarm optimization	Dosimetric	+	+	Li et al (2005)
		Integer programming	Dosimetric	+	+	Lee et al (2003)
		Response surface algorithms	Dosimetric	++	++	Aleman et al (2009)
	Local	Neighborhood search	Dosimetric	+++	+++	Aleman et al (2008), Mišić et al (2010)
		Cluster analysis	Dosimetric	+++	++	Bangert and Oelfke (2010)
		Gradient search	Dosimetric	+++	++	Craft (2007)
	Hybrid	Iterative methods	Dosimetric	++	+++	Breedveld et al (2012)
		Pattern search methods	Dosimetric	+++	++++	Rocha et al (2013b, 2016, 2019)
			Anatomical + Dosimetric	+++	++++	Rocha et al (2013a)
		Two-phase hybrid	Dosimetric	+++	++++	Lim and Cao (2012)

**A1**	Machine learning	Neural networks	Anatomical	++++	+++	Rowbottom et al (1999)
			Dosimetric	++++	+++	Dias et al (2014)

+ “Poor”, ++ “Average”, ++++ “Good”, and ++++ “Very Good”

## Appendix C. Example of minimization of maximum regret

Let us consider 9 different scenarios that consider range and positioning uncertainty. In a first stage, the optimal solutions (the optimal treatment plans) for each scenario, considering the minimization of a score, are calculated.

**Table:**

Scenario	1	2	3	4	5	6	7	8	9
Optimal score value	16, 52	16, 53	16, 52	14, 00	13, 76	13, 91	14, 85	17, 17	15, 56

The optimization algorithm will try to find a solution (a treatment plan) such that the difference between the score of this plan in each of these scenarios and the score of the corresponding optimal solution is as low as possible. Consider a solution such that, when applied to each one of the 9 scenarios, has the following scores:

**Table:**

Scenario	1	2	3	4	5	6	7	8	9
Score value	33, 64	32, 21	32, 52	33, 31	31, 76	32, 99	31, 89	33, 38	32, 77

Then, the regret in each scenario is equal to:

**Table:**

Scenario	1	2	3	4	5	6	7	8	9
Optimal score value	16, 52	16, 53	16, 52	14, 00	13, 76	13, 91	14, 85	17, 17	15, 56
Score value	33, 64	32, 21	32, 52	33, 31	31, 76	32, 99	31, 89	33, 38	32, 77
Regret	17, 12	15, 68	16	19, 31	18	19, 08	17, 04	16, 21	17, 21

This solution would generate a worst-case regret equal to 19, 31. With another solution, the situation is different:

**Table:**

Scenario	1	2	3	4	5	6	7	8	9
Optimal score value	16, 52	16, 53	16, 52	14, 00	13, 76	13, 91	14, 85	17, 17	15, 56
Score value	31, 63	29, 91	30, 43	30, 78	30, 43	30, 56	30, 06	31, 04	30, 89
Regret	15, 11	13, 38	13, 91	16, 78	16, 67	16, 65	15, 21	13, 87	15, 33

The worst regret generated by this solution is 16, 78, so this is a better solution than the previous one. The optimization algorithm will, iteratively, try to find solutions that minimize this worst (maximum) regret.
